# Coronavirus Disease (COVID-19): A Perspective from Immunosenescence

**DOI:** 10.14336/AD.2020.0831

**Published:** 2021-02-01

**Authors:** Miguel Genebat, Laura Tarancón-Díez, Rebeca de Pablo-Bernal, Alba Calderón, Mª Ángeles Muñoz-Fernández, Manuel Leal

**Affiliations:** ^1^Internal Medicine Department, Hospital Fátima, Sevilla, Spain.; ^2^Emergency Department, Virgen del Rocío University Hospital, Sevilla, Spain.; ^3^Immunology Section, Laboratorio Inmuno-Biología Molecular (LIBM), Hospital General Universitario Gregorio Marañón, Instituto de Investigación Sanitaria Gregorio Marañón (IISGM), Madrid, Spain.; ^4^Exterior Health Service, Spanish Government Delegation in the Canary Islands, Las Palmas de Gran Canaria, Spain.; ^5^Infectious Diseases and Immunology Unit, Internal Medicine Department, Hospital Viamed Santa Ángela de la Cruz, Sevilla, Spain.

Since the first reported cases with severe acute respiratory syndrome caused by a novel coronavirus (SARS-CoV2), this disease called coronavirus disease (COVID-19) has expanded worldwide being considered by World Health Organization as a pandemic. Although this virus may infect people regardless of age, race or sex, older subjects have been identified as a high-risk group regarding the clinical outcome of the disease, both for developing severe pneumonia with respiratory distress and death. Although global mortality rate directly related to SARS-CoV2 infection is unknown (a real infectious rate is not well known), mortality rate among severely elderly patients (between 60-90 years old) is around 50%, even in countries with significant lower deaths [1-2]. Hence, apart from other risk factors linked to a poor clinical outcome, such as hypertension, diabetes, cardiovascular disease, cancer or chronic lung disease [3], old age itself can be also considered as an independent risk factor associated with SARS-CoV2-related severe pneumonia and death.

From an immunopathogenic viewpoint, COVID-19 disease has probably a multifactorial nature and the final severe lung damage observed in COVID-19 could be caused by an uncontrolled proinflammatory cytokine cascade (called “cytokine storm”), driven mainly by interleukin-6 (IL-6) and other proinflammatory cytokines such as IL1β, IL8, CXCL10, and CCL2. Based on this hypothesis, apart from non-specific antiviral agents, anti-inflamatory drugs such as glucocorticoids and anti-IL6 (Tocilizumab) have been proposed to be used in patients with advanced COVID-19 disease [4]. However, which immunopathogenic status precedes this “cytokine storm” and why the elderly population is more severely affected, are currently unanswered questions. Thus, we propose that immunosenescence and age-related thymic dysfunction could play a relevant role in current COVID-19 disease scenario.

We consider the “cytokine storm” just the consequence but not the predisposing physiopathologic status that may lead to severe lung involvement, mainly in elderly subjects. Hence, an alternative immunopathogenic model is required to develop future therapeutic approaches in the scenario of COVID-19. Therefore, we suggest a new model in which immunosenescence is the underlying physiopathologic substrate that predisposes further clinical outcomes. The model is based in our knowledge and previous experience, thymic function is not only partially preserved in adult patients, but also in the scenario of chronic inflammation observed in elderly population.

## Thymic function relevance in adulthood: potential role in COVID-19 disease

**Figure 1.**
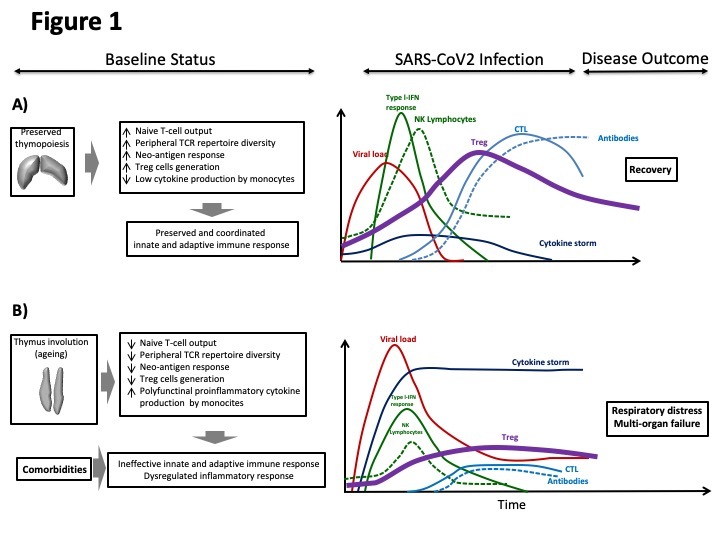
Figurative kinetics of adaptive and innate immune system response against SARS-CoV2 and disease evolution and outcome based on the degree of baseline thymopoiesis status. Left panels: baseline status for naïve T-cell output, peripheral TCR repertoire diversity, neo-antigen response, Treg-cells generation and monocyte cytokine production in patients with preserved (A) and impaired thymopoiesis and comorbidities due to ageing (B). Multiple degrees of variability can be included from A to B status. Right panels: temporal evolution for viral load, cytokine storm, type-I-IFN response, NK-lymphocyte levels, Treg cells, CTL and antibody level following exposure to SARS-CoV2 infection. Likely disease outcome is also indicated. IFN: interferon. Treg: regulatory T-cell. NK lymphocyte: Natural Killer lymphocyte. CTL: cytotoxic T lymphocyte. TCR: T-cell receptor.

Despite traditional knowledge, thymic function is at least partially preserved in adult people [5-6]. We have shown that thymic function becomes relevant regarding T-CD4 cells increase in adult HIV-infected subjects under effective antiretroviral therapy [7]. Oppositely, an impaired thymopoiesis is associated with systemic inflammation, altered T-cell homeostasis and mortality in elderly subjects [6, 8, 9]. It could also contribute to a loss of circulating lymphocyte count, especially regulatory T-cells, leading to a poor clinical outcome in the context of COVID-19 [10]. The role of thymus could also explain the less severe phenotype observed in children [11] and, taking altogether, the thymus involution and function impairment in elderly patients might contribute to a dysregulated and ineffective innate and adaptive immune response against novel antigens such as SARS-CoV2 (Fig. 1) and could explain the less severe phenotype observed in children.

In this scenario of novel SARS-CoV2 infection, a low lymphocyte count at admission, along with other pathologies such as renal failure, have been considered as a predictor factor of poor clinical outcome [12-13]. In our proposed physiopathological hypothesis, an impaired thymic function could contribute to this lymphopenia and, consequently, to a worse clinical outcome mainly in elderly subjects but also in younger patients [6, 14]. In accordance with our hypothesis, a retrospective study using thymosine-α-1 as treatment improved the clinical outcome of patients with severe COVID-19, through an enhanced thymopoiesis [15].

## Chronic systemic inflammation in elderly subjects

Low-level age-related chronic systemic inflammation (*Inflamm-aging*) is implicated in immunosenescence and atherogenesis, increasing cardiovascular morbidity. We have reported that healthy elderly subjects express high levels of proinflammatory soluble biomarkers, such as high sensitive C reactive protein, IL-8, IL-6 and TNF-α, similarly to the pattern observed once the “cytokine storm” emerges in COVID-19 severe disease, and a greater percentage of monocytes expressing proinflammatory and anti-inflammatory cytokines [16-17] compared with young subjects. Moreover, in our study, when monocytes were previously stimulated with anti-TLRs, the percentage of monocytes that responded by producing cytokines to the stimulus in the elderly group was higher and more polyfunctional, which means a higher ability to produce more than one cytokine simultaneously by monocyte.

Hence, primed monocytes (innate immunity destined cells to become macrophages in cell tissues) of old people exacerbated the pro-inflammatory profile of cytokine production, suggesting that after tissue migration these macrophages could behave similarly. It has been recently reported that patients with severe COVID-19-related lung disease have increased levels of proinflammatory cytokines, both in peripheral blood and bronchoalveolar lavage [18] and some evidence highlighted the correlation of “inflamm-aging” with increased risk of “cytokine storm” in those critical elderly patients with COVID-19 infection [19]. An experimental study performed in SARS-CoV2-infected primates showed that older animals had a higher expression of proinflammatory cytokines and a greater damaged in lung tissue compared with younger animals, with similar level of viral replication [20].

According to our dual physiopathological hypothesis, in addition to impaired thymic function previously mentioned, we believe that elderly subjects at baseline show a systemic low-level chronic inflammation. Their population of monocytes generate a great amount and variety of cytokines (multiple circulating cytokines). These cells of elderly subjects, when stimulated by pathogen-associated molecular patterns receptors like TLR by a novel agonist (SARS-CoV2 antigen), could generate the massive and polyfunctional proinflammatory cytokine release that characterized COVID-19 that would trigger the respiratory distress and multiorgan failure as clinical outcome.

Summarising, the objective of the present opinion article is to add a novel approach of a possible pathophysiology mechanisms for SARS-CoV2 infection that may explain the severity of clinical outcome observed in the COVID-19 elderly patients, but not in young patients. In our proposed model we grant a critical role to the thymus, since a preserved thymopoiesis, similar to the thymic function observed in children, could help to maintain the peripheral T-cell pool and a balanced systemic inflammation, reducing the “cytokine storm” cascade after infectious insult by SARS-CoV2.

### Author contribution

Miguel Genebat and Manuel Leal had the idea for the article, Manuel Leal and Alba Calderón performed the literature search, Rebeca de Pablo and Laura Tarancon-Diez performed the data analysis. Miguel Genebat wrote the draft of the manuscript. Mª Angeles Muñoz-Fernández, Rebeca de Pablo and Manuel Leal revised and corrected the manuscript.
